# Use of Indoor Tanning Devices by High School Students in the United States, 2009

**Published:** 2011-08-15

**Authors:** Gery P. Guy, Eric Tai, Lisa C. Richardson

**Affiliations:** Division of Cancer Prevention and Control, Centers for Disease Control and Prevention; Centers for Disease Control and Prevention, Atlanta, Georgia; Centers for Disease Control and Prevention, Atlanta, Georgia

## Abstract

The objectives of this study were to provide estimates of indoor tanning device use among US high school students and provide baseline data before implementation of a 10% excise tax on indoor tanning device use mandated by recent federal health care reform legislation. We examined the frequency of indoor tanning device use by using data from the 2009 national Youth Risk Behavior Survey. Overall, 15.6% of students used an indoor tanning device during the 12 months before the survey; almost half of those students used an indoor tanning device 10 or more times. Reported use and frequency of use varied by age, sex, and race/ethnicity. Given the high prevalence of indoor tanning device use among US high school students and the associated risk of melanoma, strategies to reduce exposure must be examined.

## Objective

The incidence of cutaneous melanoma, the most deadly form of skin cancer and one of the most commonly diagnosed cancers among adolescents, is increasing throughout the United States (1,2). Exposure to UV radiation through indoor tanning devices increases melanoma risk; exposure at an early age and frequency of use are both documented risk factors (3,4). The federal Patient Protection and Affordable Care Act, passed in 2010, includes an excise tax on indoor tanning device use. The objectives of this study were to provide estimates of indoor tanning device use among US high school students and provide baseline data before implementation of this tax.

## Methods

The 2009 Youth Risk Behavior Survey (YRBS), a component of the Youth Risk Behavior Surveillance System developed by the Centers for Disease Control and Prevention (CDC), was administered from February through May, 2009. YRBS uses a 3-stage cluster sample design to produce a nationally representative sample of public and private high school students in grades 9 through 12. Students completed the 98-item self-administered questionnaire during 1 class period and recorded their responses directly in a computer-scannable questionnaire booklet. The school response rate was 81%, the student response rate was 88%, and the overall response rate was 71%. Data from 16,410 students in 158 schools were available for this analysis. Weights based on student sex, race/ethnicity, and grade were applied to adjust for school and student nonresponse and oversampling of black and Hispanic students (5).

We assessed indoor tanning device use with the question "During the past 12 months, how many times did you use an indoor tanning device such as a sunlamp, sunbed, or tanning booth? (Do not include getting a spray-on tan.)" Response choices were 0 times, 1 or 2 times, 3 to 9 times, 10 to 19 times, 20 to 39 times, or 40 or more times. We calculated prevalence estimates of using an indoor tanning device at all (ie, 1 or more times during the 12 months before the survey) and the frequency of use (ie, 1-9 times, 10 or more times) and their corresponding 95% confidence intervals overall and by age, sex, and race/ethnicity. We generated all point estimates and confidence intervals using weighted data, and used survey data commands (svy) in STATA (StataCorp LP, College Station, Texas) to account for the complex survey design. We used the Pearson χ^2^ test to examine associations between indoor tanning device use and demographic characteristics.

## Results

Nationwide, 15.6% of students used an indoor tanning device 1 or more times during the 12 months before the survey (Table 1). Overall, 7.9% of students used an indoor tanning device 1 to 9 times, and 7.7% used an indoor tanning device 10 or more times. Use of indoor tanning devices 1 or more times during the 12 months before the survey varied significantly by age, sex, and race/ethnicity; the prevalence was higher among older students, female students, and white students.

Among students reporting the use of indoor tanning devices during the 12 months before the survey, 49.1% did so 10 or more times (Table 2). Frequency of use in this group varied significantly by age, sex, and race/ethnicity. For example, among students who reported indoor tanning device use in the 12 months before the survey, female students and white students were more likely to use such services 10 or more times.

## Discussion

Our results indicate that indoor tanning device use is widespread among US high school students, and the use of indoor tanning devices multiple times is common. This study is consistent with previous reports showing widespread use of indoor tanning devices among adolescents and a higher prevalence among older adolescents, females, and whites (6,7).

Given the known health risks associated with indoor tanning device use, many health-related organizations recommend regulations limiting minors' access to these devices (8,9). In the United States, 26 states have laws restricting minors' access to tanning facilities; statutory requirements include parental consent or accompaniment, physician authorization, and bans on tanning device use (10). However, the presence of state legislation restricting minors' access to indoor tanning devices has limited effectiveness (7). Our results suggest that additional strategies for reducing indoor tanning device use among US high school students need to be examined. Such efforts could include public education about the risks of indoor tanning device use, efforts aimed at changing the social norms regarding skin tanning, better enforcement of current restrictions, and more stringent legislative measures prohibiting the use of indoor tanning devices among minors.

This study is subject to certain limitations. The results from this study are generalizable only to youths who attend high school and may not be representative of the entire youth population. Additionally, the use of an indoor tanning device was self-reported, and the degree of misreporting cannot be determined. Although reliability data are not available for the question about indoor tanning device use, a previous reliability study of the YRBS questionnaire demonstrated most items had good test-retest reliability (11). An important strength of this study is that it provides a nationally representative estimate of indoor tanning device use among US high school students.

The Patient Protection and Affordable Care Act includes a 10% excise tax on indoor tanning device use. This newly enacted tax may reduce the use of indoor tanning devices, especially among adolescents. A 10% increase in the price of tobacco led to an approximate 4% reduction in tobacco use prevalence among adolescents (12); thus, the newly imposed tax may reduce indoor tanning device use and UV radiation exposure, a documented risk factor for melanoma. Because this study provides nationally representative estimates, it can serve as a baseline for continued monitoring of indoor tanning device use and evaluating the effect of the new excise tax and other interventions on reducing indoor tanning device use among US high school students.

## Figures and Tables

**Table 1:** Indoor Tanning Device Use^a^ Among US High School Students, Youth Risk Behavior Survey, 2009

Characteristic	n	Reported Use, % (95% CI)	No. of Times Used in Previous 12 Months, % (95% CI)				

			1-2	3-9	10-19	20-39	≥40
**Total**	14,590	15.6 (13.7-17.6)	3.6 (3.1-4.2)	4.3 (3.8-4.9)	2.9 (2.3-3.6)	2.4 (2.0-2.9)	2.4 (2.0-2.9)
**Age, y**							
≤14	1,471	9.7 (7.7-12.2)	2.5 (1.6-3.9)	2.5 (1.8-3.6)	1.8 (1.1-3.0)	1.5 (0.9-2.5)	1.4 (0.9-2.2)
15	3,287	12.0 (10.1-14.1)	3.5 (2.8-4.4)	3.4 (2.7-4.3)	2.3 (1.7-3.2)	1.3 (0.9-1.9)	1.4 (1.0-1.9)
16	3,705	14.9 (12.7-17.4)	3.8 (3.0-4.7)	4.2 (3.4-5.2)	2.5 (2.0-3.3)	1.9 (1.4-2.6)	2.5 (1.7-3.6)
17	3,755	19.1 (16.8-21.7)	3.7 (3.0-4.7)	4.8 (3.9-5.9)	3.6 (2.6-5.0)	3.4 (2.7-4.2)	3.5 (2.7-4.6)
≥18	2,305	22.0 (19.0-25.4)	4.2 (3.2-5.4)	6.7 (5.2-8.5)	4.2 (3.0-5.7)	4.0 (3.0-5.3)	3.0 (2.2-4.1)
**Sex**							
Female	7,314	25.4 (22.4-28.6)	5.4 (4.7-6.1)	6.9 (6.0-8.0)	5.1 (4.0-6.4)	4.4 (3.6-5.3)	3.7 (2.9-4.6)
Male	7,219	6.7 (5.6-8.0)	2.1 (1.6-2.6)	2.0 (1.6-2.4)	0.9 (0.6-1.4)	0.6 (0.4-0.8)	1.3 (0.9-1.8)
**Race/ethnicity**							
White	6,606	21.1 (18.3-24.2)	4.5 (3.8-5.3)	5.9 (5.2-6.6)	4.1 (3.3-5.3)	3.4 (2.7-4.2)	3.2 (2.6-3.9)
Black	2,405	4.5 (3.1-6.4)	1.4 (0.9-2.1)	1.0 (0.6-1.5)	0.6 (0.3-1.1)	0.8 (0.4-1.5)	0.8 (0.5-1.3)
Hispanic	3,985	8.2 (6.9-9.7)	3.3 (2.6-4.2)	2.0 (1.5-2.8)	1.0 (0.7-1.6)	0.6 (0.4-1.1)	1.1 (0.8-1.6)

Abbreviation: CI, confidence interval.

a Defined as using a sunlamp, sunbed, or tanning booth (not including a spray-on tan). Estimates based on weighted data. Sample sizes are unweighted and may not add to total because of missing data. Reported use of indoor tanning devices ≥1 time during the 12 months before the survey varied significantly by age, sex, and race/ethnicity (*P* < .001 for all).

**Table 2:** Frequency of Indoor Tanning Device Use^a^ Among US High School Students Who Used an Indoor Tanning Device at Least Once in Previous 12 Months, Youth Risk Behavior Survey, 2009

**Characteristic**	n	1-9 Times, % (95% CI)	≥10 Times, % (95% CI)	*P* Value
**Total**	2,026	50.9 (47.4-54.4)	49.1 (45.6-52.6)	NC
**Age, y**				
≤14	150	52.0 (43.3-60.6)	48.0 (39.4-56.7)	.005
15	390	57.9 (52.6-63.1)	42.1 (36.9-47.4)	
16	464	53.6 (47.8-59.4)	46.4 (40.6-52.2)	
17	598	44.9 (39.9-49.9)	55.1 (50.1-60.1)	
≥18	415	49.4 (42.7-56.1)	50.6 (43.9-57.3)	
**Sex**				
Female	1,517	48.3 (44.3-52.4)	51.7 (47.6-55.7)	.02
Male	500	59.9 (52.0-67.3)	40.1 (32.7-48.0)	
**Race/ethnicity**				
White	1,469	49.0 (45.5-52.6)	51.0 (47.4-54.5)	<.001
Black	113	52.8 (42.6-62.7)	47.2 (37.3-57.4)	
Hispanic	303	65.4 (58.1-72.0)	34.6 (28.0-41.9)	

Abbreviations: CI, confidence interval; NC, not calculated.

a Indoor tanning device use defined as using a sunlamp, sunbed, or tanning booth (not including a spray-on tan). Estimates based on weighted data. Sample sizes are unweighted and may not add to total because of missing data.
